# Supplementary material for: Current approaches to acute bronchiolitis management among pediatricians: guideline adherence and practice variation
Source: BMC Pediatr. 2026 Jul 7;26:979. doi: 10.1186/s12887-026-07285-5 (PMC13656603; doi:10.1186/s12887-026-07285-5)
Supplement: Supplementary file 3 [file 12887_2026_7285_MOESM1_ESM.docx]

**CASE REPORT FORM**

1. How old are you?
2. What is your gender?
   a. Woman
   b. Man
3. What is your medical title?
a. Pediatric resident
b. Pediatrician
c. Pediatrician, subspecialist
4. Which hospital do you work at?
a. State Hospital
b. Training and Research Hospital
c. University Hospital
d. Other
5. What is your years in the profession (year)?
6. What is the area you most frequently work in?
a. Children's outpatient clinic
b. Children's ward
c. Children's emergency room
d. Pediatric intensive care unit
e. Neonatal intensive care unit
7. How many cases of acute bronchiolitis do you see in a month?
8. After taking the history and performing the physical examination, which tests would you order to diagnose bronchiolitis? (You can select more than one option)
   a. I do not use diagnostic tests
   b. Complete blood count
   c. Acute phase reactants
   d. Blood gas
   e. Posteroanterior chest X-ray
   f. Respiratory viral pathogens
   g. Influenza rapid test
   h. Respiratory Syncytial Virus rapid test
9. Do you use the bronchiolitis score in the diagnosis and monitoring of bronchiolitis?

Yes	No

10. Do you use guidelines in the diagnosis and monitoring of bronchiolitis? Yes	No
11. Which guidelines do you use for the treatment of bronchiolitis?
12. To the emergency room What are your treatment preferences for a patient presenting with **moderate bronchiolitis ? (You can select more than one option)**
    a. Oxygen
    b. Hydration
    c. Nebulized salbutamol
    d. Nebulized epinephrine
    e. Nebulized hypertonic saline
    f. Systemic steroids
    g. HFNC
    h. nCPAP
13. To the emergency room, what are your treatment preferences for a patient presenting with **severe bronchiolitis ? (You can select more than one option)**
    a. Oxygen
    b. Hydration
    c. Nebulized salbutamol
    d. Nebulized epinephrine
    e. Nebulized hypertonic saline
    f. Systemic steroids
    g. HFNC
    h. nCPAP
14. In the children's ward, what are your treatment preferences for a patient presenting with **moderate bronchiolitis? (You can select more than one option)**
    a. Oxygen
    b. Hydration
    c. Nebulized salbutamol
    d. Nebulized epinephrine
    e. Nebulized hypertonic saline
    f. Systemic steroids
    g. HFNC
    h. nCPAP
15. In the children's ward, what are your treatment preferences for a patient presenting with **severe bronchiolitis ? (You can select more than one option)**
    a. Oxygen
    b. Hydration
    c. Nebulized salbutamol
    d. Nebulized epinephrine
    e. Nebulized hypertonic saline
    f. Systemic steroids
    g. HFNC
    h. nCPAP
