# Supplementary material for: Current approaches to acute bronchiolitis management among pediatricians: guideline adherence and practice variation
Source: BMC Pediatr. 2026 Jul 7;26:979. doi: 10.1186/s12887-026-07285-5 (PMC13656603; doi:10.1186/s12887-026-07285-5)
Supplement: Supplementary file 5 [file 12887_2026_7285_MOESM5_ESM.docx]

**Current Approaches to Acute Bronchiolitis Management Among Pediatricians: Guideline Adherence and Practice Variation**

Özlem Üzüm^1^, Gülberat Totur^2^, Kayı Eliaçık^2^, Ali Kanık^3^

^1^Dokuz Eylül Üniversity, Faculty of Medicine, Department of Pediatric Diseases, Izmir, Turkiye

^2^ University of Health Sciences, Tepecik Training and Research Hospital, Department of Social Service, Izmir, Turkey

^3^ Katip Çelebi University Faculty of Medicine, Department of Pediatric Diseases, Izmir, Turkey

Özlem Üzüm, Associate Professor, Dokuz Eylül Üniversity, Faculty of Medicine, Department of Pediatric Diseases, İzmir, Türkiye, <https://orcid.org/0000-0003-3297-7476>

Gülberat Totur, Medical Doctor, Ministry of HealthSciences University, Tepecik Education and Research Hospital, Department of Pediatric Diseases, Izmir, Turkey, https://orcid.org/[0000-0002-1845-4161](https://orcid.org/0000-0002-1845-4161)

Kayı Eliaçık, Professor, Ministry of HealthSciences University, Tepecik Education and Research Hospital, Department of Pediatric Diseases, Izmir, Turkey, [https://orcid.org/0000-0001-9529-9719](https://orcid.org/0000-0003-3297-7476)

Ali Kanık, Professor, Katip Çelebi University Faculty of Medicine, Department of Pediatric Diseases, Izmir, Turkey, [https://orcid.org/0000-0003-3304-0992](https://orcid.org/0000-0003-3297-7476)

**Corresponding Author**

Özlem ÜZÜM, Assoc. Prof

Dokuz Eylül Üniversity, Faculty of Medicine, Department of Pediatric Diseases,

Izmir, Turkiye

ORCHID ID: 0000-0003-3297-7476

E-mail: baspinarozlemm@hotmail.com

Mobile: +90 507 785 96 37

**Author Contributions**

ÖÜ wrote the original manuscript, derived the models, and GT analyzed the data. ÖÜ, GT, and KE reviewed the final manuscript in consultation with AK, and ÖÜ and GT reviewed the original manuscript, edited the style and English, and provided opinions in discussion and analysis. KE and AK supervised the study, administering the research planning and execution. All authors equally contributed to the result evaluation and manuscript review before submission.

**Acknowledgments**

None.

**Declaration of Conflicting Interests**

The author(s) declared no potential conflicts of interest with respect to the research, authorship, and/or publication of this article.

**Funding**

The author(s) received no financial support for the research, authorship, and/or publication of this article.

**Ethical Approval**

The study was approved by the ethics committee of Tepecik Training and Research Hospital on 08/07/2022, with protocol number 2022/07-19. All procedures performed in this study involving human participants followed the institutional research committee’s ethical standards and the 1964 Helsinki Declaration and its later amendments or comparable ethical standards. Informed consent was obtained from all participants.

**Consent to Participate**

All participants gave their consent.

**Consent for Publication**

Not Applicable.

**Availability of Data and Materials**

The datasets used and/or analysed during the current study are available from the corresponding author on reasonable request.

**Current Approaches to Acute Bronchiolitis Management Among Pediatricians: Guideline Adherence and Practice Variation**

**Abstract**

**Background:** Although evidence-based clinical guidelines recommend supportive care as the mainstay of acute bronchiolitis management, significant variability in diagnostic and therapeutic practices among pediatricians persists. Identifying factors associated with guideline adherence may help reduce the gap between evidence-based recommendations and real-world practice.

**Methods:** This cross-sectional study was conducted between January and December 2023 using a structured, web-based questionnaire administered to pediatricians working in different clinical settings. Data were collected on demographic characteristics, use of bronchiolitis severity scoring systems, adherence to clinical guidelines, diagnostic and treatment preferences for moderate and severe bronchiolitis in pediatric emergency departments and wards. Participants were categorized based on guideline adherence and professional status (resident or specialist).

**Results:** A total of 239 pediatricians participated. 38.1% of pediatricians reported routine use of a bronchiolitis severity scoring system, while 59.8% reported adherence to at least one clinical guideline. Guideline adherence was significantly higher among residents and among those using severity scoring systems. Oxygen and hydration were the most commonly preferred across all settings. The use of nebulized therapies and systemic steroids increased with disease severity, despite limited recommendations in guidelines. Non-recommended therapies remained frequently used, particularly in severe cases. Pediatricians adhering to guidelines showed a stronger preference for supportive care, whereas non-adherent pediatricians more often favored nebulized bronchodilators. Especially in cases of moderate acute bronchiolitis, pediatricians adhering to guidelines used oxygen significantly more frequently (p=0.012), while those who did not adhere used salbutamol significantly more frequently (p<0.005).

**Conclusions:** Considerable variability remains in the management despite established clinical guidelines. Disease severity, professional experience, and use of severity scoring systems influence adherence to evidence-based practices. Targeted educational interventions may help improve guideline implementation and promote supportive, high-value care in pediatric settings.

**Keywords:** acute bronchiolitis, guideline adherence, pediatricians, practice variation, supportive care

**Current Approaches to Acute Bronchiolitis Management Among Pediatricians: Guideline Adherence and Practice Variation**

**Background**

Acute bronchiolitis is a common lower respiratory tract infection affecting children under two years of age and represents one of the leading causes of emergency department visits and hospitalizations in infancy. Diagnosis is primarily based on clinical history and physical examination, and routine laboratory testing or imaging is generally not recommended [1,2]. Clinical severity is typically classified as mild, moderate, or severe, and severity scoring systems may be used to support clinical decision-making regarding patient monitoring and disposition [1,3,4].

To promote standardized and evidence-based management, several national and international clinical practice guidelines—most notably those published by the American Academy of Pediatrics (AAP) and the National Institute for Health and Care Excellence (NICE) have been developed and periodically updated [1,2,5]. These guidelines consistently emphasize supportive care, including oxygen therapy and hydration, while discouraging the routine use of diagnostic tests and pharmacological treatments such as bronchodilators, nebulized epinephrine, and systemic steroids. Individual institutions often adapt these recommendations to local protocols, integrating guideline principles with clinical experience.

Despite the availability of well-established guidelines, variations in the management of acute bronchiolitis persist [1,2,6]. In particular, nebulized therapies such as salbutamol, epinephrine, hypertonic saline, and systemic steroids remain widely used in clinical practice, even though their routine use is not supported by strong evidence [7-9]. These variations may reflect differences in clinical training, professional experience, institutional culture, and perceptions of disease severity. As a result, real-world practice may diverge from guideline recommendations, especially in more severe cases or high-pressure clinical settings.

Previous studies evaluating diagnostic and therapeutic approaches in acute bronchiolitis have largely focused on patient outcomes or comparisons between viral etiologies [3,6]. However, fewer studies have specifically examined pediatricians’ adherence to clinical guidelines and the factors associated with variability in practice patterns. Understanding how pediatricians interpret and implement guideline recommendations in daily practice may help identify existing gaps between evidence-based guidance and real-world management.

Therefore, this study aimed to evaluate pediatricians' current approaches to the management of acute bronchiolitis, with a particular focus on guideline adherence. We sought to examine the use of severity scoring systems, diagnostic testing, and treatment preferences across clinical settings and disease severity levels, as well as to explore differences by professional status. By identifying practice patterns associated with guideline adherence, this study aims to highlight areas where targeted educational strategies may improve the implementation of evidence-based care.

**Materials and Methods**

This cross-sectional study was conducted between January and December 2023 using a structured, web-based questionnaire administered to pediatricians working in different clinical settings. Eligible participants included general pediatrics residents, general pediatrics specialists, subspecialty residents, and subspecialty specialists. There are approximately 10,000 pediatric physicians and residents in our country [10]. It was aimed to reach 370 physicians with a 95% confidence level and 5% margin of error, and the survey was distributed via online platforms (SurveyMonkey and Google Forms), and participation was voluntary. However, 239 pediatricians completed the survey within the specified period. All pediatricians who consented to participate in the study completed the questionnaires in full. Data were collected using a structured questionnaire developed by the researchers specifically for this study. An English version of this survey is available as a supplementary file.

The study was conducted in accordance with the principles of the Declaration of Helsinki. Ethical approval was obtained from the Local Ethics Committee of Tepecik Training and Research Hospital before data collection, and electronic informed consent was obtained from all participants before completion of the questionnaire (08/07/2022, Decision No: 2022/07-19). Informed consent was obtained from all participants.

The questionnaire collected data on demographic and professional characteristics, including age, sex, type of institution, years of medical practice, and the approximate monthly number of acute bronchiolitis cases managed by the participant.

Participants were asked about their use of clinical practice guidelines for the diagnosis and management of acute bronchiolitis, including whether they followed any national or international guidelines and whether they used an acute bronchiolitis severity-scoring system in the pediatric emergency department (PED) and pediatric ward (PW).

Diagnostic approaches evaluated in the survey included the use of complete blood count, acute-phase reactants, blood gas analysis, posteroanterior chest radiography, respiratory viral pathogen testing, rapid influenza testing, and rapid respiratory syncytial virus (RSV) testing.

Treatment preferences were assessed separately for patients with moderate and severe acute bronchiolitis managed in the PED and in the PWd. Treatment modalities evaluated included oxygen therapy, hydration, nebulized salbutamol, nebulized epinephrine, nebulized hypertonic saline, systemic steroids, and advanced respiratory support methods such as high-flow nasal cannula (HFNC) and nasal continuous positive airway pressure (nCPAP).

Participants who reported using at least one clinical guideline for the management of acute bronchiolitis were classified as guideline-adherent physicians (Group 1). Those who reported not using clinical guidelines or who considered guidelines unnecessary were classified as non-adherent physicians (Group 2).

In addition, pediatricians were categorized according to professional status as resident pediatricians or specialist pediatricians (including general pediatrics specialists and subspecialty specialists) to evaluate differences in guideline adherence, diagnostic approaches, and treatment preferences.

***Statistical Analysis:*** Categorical variables were summarized as frequencies and percentages. Comparisons between groups were performed using the chi-square test or Fisher’s exact test, as appropriate, based on cell counts. To identify the independent predictors of clinical guideline non-adherence among participating pediatricians, a multivariable binary logistic regression analysis was performed. Demographic traits, institutional types, and clinical preferences were simultaneously entered into the model as independent covariates. Adjusted Odds Ratios (ORs) and their corresponding 95% Confidence Intervals (CIs) were calculated. All statistical analyses were conducted using IBM SPSS Statistics version 24 (IBM Corp., Chicago, IL, USA). A two-sided p-value of <0.05 was considered statistically significant.

**Results**

A total of 239 pediatricians completed the survey. The mean age of participants was 36.6±6.4 years, and 69.5% were female. Most respondents were specialists working in training and research hospitals. The median duration of medical practice was 10 years, and participants reported managing a median of 42 acute bronchiolitis cases per month across emergency department and outpatient settings (Table 1).

38.1% of pediatricians reported routine use of a bronchiolitis severity scoring system, while 59.8% reported adherence to at least one clinical guideline. Guideline adherence was significantly higher among pediatricians who reported using a severity scoring system compared with those who did not (p<0.005) (Table 2). When stratified by professional status, residents demonstrated significantly higher guideline adherence than specialists, whereas use of severity-scoring systems was similar between groups (p=0.001) (Table 3).

During the diagnostic evaluation of acute bronchiolitis, 38.1% of pediatricians reported not requesting any diagnostic tests. Among those who did request investigations, complete blood count was the most frequently ordered test, followed by posteroanterior chest radiography and acute-phase reactants (Table 2).

There were no significant differences between guideline-adherent and non-adherent pediatricians in the use of complete blood count, blood gas analysis, chest radiography, or respiratory viral pathogen testing. However, acute-phase reactant testing was significantly more common among non-adherent pediatricians (p=0.013) (Table 2). Respiratory viral pathogen testing was significantly more common among resident pediatricians than pediatric specialists (p=0.006) (Table 3).

Across all clinical settings, oxygen therapy and hydration support were identified as the most frequently preferred treatment modalities in the total study population. Specifically, overall preference rates ranged from 83.3% to 100% for oxygen therapy and 78.7% to 94.1% for hydration across the groups. Furthermore, the utilization rates of nebulized therapies and systemic steroids demonstrated a clear upward trend with worsening clinical severity in both settings: In the PED, as disease severity advanced from moderate to severe, the overall use of nebulized salbutamol increased from 72.4% to 82.0%, nebulized epinephrine rose from 12.6% to 28.5%, and systemic steroids increased sharply from 34.3% to 71.1%.In the PW, similarly, moving from moderate to severe disease, the overall preference for nebulized salbutamol rose from 74.1% to 83.3%, nebulized epinephrine from 8.8% to 26.4%, and systemic steroids escalated from 43.5% to 69.9% (Table 2).

When treatment preferences were compared according to guideline adherence, pediatricians in the guideline-adherent group demonstrated a significantly higher use of specific supportive therapies: oxygen therapy for moderately ill patients in both the PED and PW; hydration support exclusively for moderately ill patients in the PED; HFNC for severely ill patients in the PW; and nCPAP for both moderately and severely ill patients across both the PED and PW settings (Table 2). Finally, a multivariable binary logistic regression analysis was performed to identify the independent predictors of clinical guideline non-adherence among the participating pediatricians. The model simultaneously adjusted for demographic characteristics, professional titles, institutional settings, and clinical preferences. The regression analysis revealed that demographic features (age, sex, and years of practice) and professional titles were not independent predictors of guideline non-adherence. Instead, specific diagnostic and therapeutic choices were identified as independent predictors. Requesting acute-phase reactants (p=0.020, OR=0.224), utilizing nebulized salbutamol (p=0.005, OR=0.144), and using HFNC (p=0.040, OR=0.110) for moderately ill patients in the PED setting were independently associated with guideline non-adherence. In the PW, specific preferences for advanced respiratory support modalities in severely ill patients, including HFNC (p=0.008, OR=7.661) and nCPAP (p=0.024, OR=6.148), also demonstrated a strong and independent statistical association with guideline non-adherence (Table 4).

When treatment preferences were evaluated based on professional title, resident pediatricians demonstrated a significantly higher preference for nebulized salbutamol in moderately ill patients in the PED, HFNC in severely ill patients in the PED, nCPAP in moderately ill patients in the PW, and HFNC in severely ill patients in the PW. Conversely, specialist pediatricians showed a significantly higher use of nebulized epinephrine for moderately ill patients in the PED, as well as nebulized salbutamol, epinephrine, and nCPAP for severely ill patients in the PED. Additionally, specialists more frequently preferred systemic steroids for moderately ill patients in the PW and nebulized epinephrine for severely ill patients in the PW (Table 3).

**Discussion**

In this study, we evaluated current approaches to the management of acute bronchiolitis among pediatricians, with a particular focus on guideline adherence, diagnostic practices, and treatment preferences across different clinical settings and disease severities. Our findings demonstrate that although clinical guidelines for acute bronchiolitis are widely available and recognized, substantial variability persists in real-world practice. Importantly, pediatricians who reported using severity scoring systems and those who adhered to clinical guidelines were more likely to follow supportive, guideline-recommended management strategies.

However, it is critical to interpret these adherence rates within the context of our study's criteria. We defined guideline adherence based on the self-reported utilization of at least one clinical guideline. While this inclusive definition was chosen to capture broad baseline awareness, it admittedly represents a relatively low methodological threshold. Consequently, this threshold may have inflated the documented adherence rates, classifying some clinicians as "adherent" based on their general familiarity, while potentially masking significant deviations from core recommendations in real-world practice, such as the over-prescription of non-recommended nebulized or systemic therapies.

One of the key findings of this study is the strong association between the use of acute bronchiolitis severity scoring systems and guideline adherence. Pediatricians who incorporated scoring systems into their clinical practice demonstrated significantly higher adherence to guideline recommendations. This finding is consistent with previous studies suggesting that structured clinical assessment tools may support more standardized decision-making and reduce unnecessary interventions [11,12]. Severity scoring systems may function not only as tools for patient assessment but also as cognitive frameworks that reinforce evidence-based management principles during clinical decision-making.

Despite clear guideline recommendations discouraging routine diagnostic testing in acute bronchiolitis, a substantial proportion of pediatricians in this study continued to request laboratory and imaging studies. Although more than one-third of participants reported not ordering any diagnostic tests, complete blood count, chest radiography, and acute-phase reactants remained commonly used. Similar patterns have been reported in previous studies, highlighting the persistent gap between guideline recommendations and clinical practice [3,7,13,14]. The higher frequency of acute-phase reactant testing among non-adherent pediatricians may reflect diagnostic uncertainty or a perceived need to exclude bacterial infection, despite limited evidence supporting the clinical utility of these tests in routine bronchiolitis management. Our multivariable logistic regression analysis further strengthened this finding by demonstrating that requesting AFR remains an independent behavioral predictor of guideline non-adherence. This indicates that even when adjusting for potentially confounding variables such as the physician’s age, years of practice, or institutional settings, the defensive reflex to order non-recommended inflammatory markers strongly characterizes clinicians who stray from evidence-based protocols. This behavior typically stems from diagnostic uncertainty or the perceived pressure to rule out secondary bacterial infections, a clinical trend extensively documented in broader pediatric respiratory literature.

The identification of viral pathogens, including RSV and influenza, was infrequently requested by pediatricians in this study. Current guidelines recommend viral testing primarily in specific clinical scenarios, such as during influenza season or for determining eligibility for RSV prophylaxis [1,15]. The low rate of viral testing observed in our study is therefore largely consistent with guideline recommendations. However, previous studies comparing RSV and non-RSV bronchiolitis have reported conflicting results regarding disease severity and clinical outcomes [3,6,16,17]. In recent post-pandemic studies, rhinovirus has emerged as a frequent causative agent, raising questions about whether pathogen-specific approaches may have future clinical relevance. Nevertheless, at present, routine viral testing is not recommended, and our findings support this conservative diagnostic approach.

With respect to treatment practices, supportive care, particularly oxygen therapy and hydration, was the most frequently preferred management strategy across all clinical settings. This aligns with current evidence emphasizing minimal intervention and avoidance of unnecessary treatments in infants with acute bronchiolitis [1,2,10,18]. However, as disease severity increased, the use of nebulized therapies also increased substantially, even among pediatricians who reported adherence to clinical guidelines. This trend suggests that clinical severity may act as a strong driver of deviation from guideline recommendations, possibly reflecting clinician discomfort, parental expectations, or the perceived need to offer active interventions in more severe cases [19].

Nebulized salbutamol and epinephrine, as well as systemic steroids, although not routinely recommended by current clinical guidelines, continued to be widely used, particularly in patients with severe disease. Previous randomized controlled trials and meta-analyses have demonstrated that these therapies may lead to transient improvements in clinical signs; however, they do not result in meaningful benefits in clinically important outcomes such as length of hospital stay or need for intensive care [7,11,20,21]. Several studies have emphasized that the management of acute bronchiolitis is influenced by pathogen characteristics, host immune responses, and their complex interactions [10,22-24]. Short-term improvements in auscultatory findings or symptoms may therefore contribute to the continued use of these therapies, despite their limited long-term efficacy. In contrast, nebulized hypertonic saline, although removed from several treatment protocols, remains conditionally recommended in some guidelines for hospitalized patients [1,2,8]. In our study, hypertonic saline was more frequently preferred by guideline-adherent pediatricians in the ‘Severely ill in PW’ setting. Differences in management approaches were also observed between resident and specialist pediatricians. Residents demonstrated higher overall guideline adherence and were more likely to prefer supportive and non-invasive respiratory support strategies, such as HFNC and nCPAP. These findings may reflect the impact of ongoing training, recent exposure to updated guidelines, and closer supervision in academic settings [11]. Within the specific context of the Turkish medical education system, several structural factors may further explain this disparity. In recent years, the Board of Specialization in Medicine (TUKMOS) in Turkey has strictly standardized the pediatric residency core curriculum, placing a heavy emphasis on evidence-based algorithms. Furthermore, the increasing momentum of institutional accreditation processes across pediatric departments in Turkey mandates strict compliance with local and international clinical pathways. Because resident pediatricians practice under continuous, multi-layered supervision by senior residents and faculty members, particularly during structured daily attending rounds, their clinical autonomy is naturally restricted, leaving less room for deviation from established protocols. In contrast, specialist pediatricians often practice with greater clinical autonomy, especially in secondary care public hospitals or private settings. Over time, specialists may become more prone to relying on entrenched personal clinical experience, local traditions, or defensive medicine practices to manage parental anxiety, leading to a higher rate of non-recommended interventions [19,21, 25]. Crucially, our multivariable model unveiled institutional type as a pivotal structural driver of protocol compliance (p=0.043). Pediatricians practicing in specific institutional settings, particularly in secondary care state hospitals or university hospitals, exhibited a staggering 6.0-fold (OR=6.021) and 16.6-fold (OR=16.677) higher likelihood of guideline non-adherence, respectively, compared to those working in training and research hospitals, which served as the reference baseline (Table 4). This independent association underscores that practice variation is not merely an individual clinician's choice but is deeply embedded within institutional cultures, local peer habits, and varying levels of administrative oversight. While academic university clinics often mandate adherence through strict, peer-reviewed clinical pathways, other healthcare facilities may lack integrated clinical decision-support systems, thereby allowing greater reliance on legacy habits or subjective clinical autonomy.

The increasing use of advanced respiratory support methods, particularly HFNC, with worsening disease severity observed in this study, is consistent with current literature. HFNC is often preferred over nCPAP due to better patient tolerance, ease of use, and a lower perceived risk of complications [26]. Our findings support the growing role of HFNC as a preferred non-invasive respiratory support modality in severe acute bronchiolitis.

Interestingly, when all therapeutic options were simultaneously analyzed in our multivariable regression model, several factors that showed high significance in the univariate analysis, such as systemic steroid use in the PED or salbutamol use in the PW, lost their independent predictive power (p≥0.05). This statistical shift suggests that these non-recommended interventions do not occur in isolation; rather, they are confounded by broader, institutional management patterns. Conversely, initiating nebulized salbutamol for moderately ill patients in the PED emerged as a robust, isolated hallmark of guideline non-adherence (p=0.005, OR=0.144). Similarly, in the PW, the conditional preference for nebulized hypertonic saline in severe cases, alongside advanced respiratory strategies like HFNC and nCPAP, stood out as independent discriminators of management stratification. These strong independent odds ratios validate that advanced respiratory care deployment and persistent bronchodilator trials are the exact clinical pivot points where guideline adherence and real-world practice divergence decisively part ways.

Limitations of the Study: This study has several limitations that should be considered when interpreting the findings. First, the data were self-reported, which may not fully capture actual clinical practices. For instance, physicians might report strict adherence to clinical guidelines, yet their actual treatment recommendations in practice could vary. Related to this, the study may be subject to social desirability bias, as pediatricians might naturally overreport their adherence to established guidelines to align with professional expectations. Furthermore, evaluating physicians' clinical approaches on a large scale heavily relies on self-reported data, making this approach a standard and necessary methodological choice for this type of research. Second, this study focused strictly on the reported use of guidelines but did not objectively assess the participants' actual knowledge, comprehension, or correct application of these guidelines. Third, the cross-sectional design of the study precludes causal inference and prevents the evaluation of temporal trends over time. Lastly, the study was conducted within a single-country setting, which may limit the generalizability of the findings to other healthcare systems with different training structures and clinical protocols. This limitation to generalizability is further compounded by the fact that our final sample (n=239) fell short of our initial target size (n=370), introducing a potential selection bias. Nevertheless, the relatively large sample size and the successful inclusion of pediatricians and residents from diverse clinical settings across the nation strengthen the relevance and regional representativeness of our findings.

In conclusion, our results demonstrate that although clinical guidelines for acute bronchiolitis are widely recognized, significant gaps persist between the recommended and actual practices. The use of severity scoring systems and professional training status appears to influence guideline adherence, while increasing disease severity remains a major driver of deviation from evidence-based management. Targeted educational interventions focusing on guideline implementation and severity-based decision-making may help reduce practice variability and promote high-value, supportive care in children with acute bronchiolitis.

**References**

1. Ralston SL, Lieberthal AS, Meissner HC, et al. Clinical Practice Guideline: The Diagnosis, Management, and Prevention of Bronchiolitis. Pediatric 2014;134:1474-502.
2. Ricci V, Delgado Nunes V, Murphy MS, Cunningham S. Guideline Development Group and Technical Team. Bronchiolitis in children: summary of NICE guidance. BMJ. 2015;350:2305.
3. Üzüm Ö, Kanık A, Eliaçık K, et al. Comparison of clinically related factors and treatment approaches in patients with acute bronchiolitis. Turk Pediatri Ars.2020;55:376–85.
4. Mahajan V, Tıwarı M, Arya A, Tıwarı A, Chawla D, Saını SS. Clinical predictors of hospital admission in acute lower respiratory tract infection in 2-month-to-2-year-old children. Respirology. 2016;21:350–6.
5. Kocabaş E, Tanır G, Cinel G. Çocuklarda Toplumda Gelişen Pnömoni Tanı, Tedavi ve Uzlaşı Raporu 2023.
6. Hon KL, Leung AKC, Wong AHC, Dudi A, Leung KKY. Respiratory Syncytial Virus is the Most Common Causative Agent of Viral Bronchiolitis in Young Children: An Updated Review. Current Pediatric Reviews. 2023; 19(2):139-49.
7. Wainwright C, Altamirano L, Cheney M, et al. A Multicenter, Randomized, Double-Blind, Controlled Trial of Nebulized Epinephrine in Infants with Acute Bronchiolitis. New Eng J of Med. 2003;349:27-35.
8. Zhang L, Mendoza-Sassi RA, Wainwright CE, Aregbesola A, Klassen TP. Nebulised hypertonic saline solution for acute bronchiolitis in infants. Cochrane Database of Systematic Reviews 2023, Issue 4. Art. No.: CD006458.
9. Song Y, Li R. Effect of budesonide combined with salbutamol nebulization on pulmonary function and serum immune factors in children with bronchiolitis. Am J Transl Res. 2021;13:8158-64.
10. Republic of Turkey Ministry of Health, General Directorate of Health Information Systems, 2024. Health Statistics Yearbook 2023. Ankara: Republic of Turkey Ministry of Health.
11. Virgili F, Nenna R, Di Mattia G, et al. Acute Bronchiolitis: The Less, the Better? Curr Pediatr Rev. 2024;20(3):216-23.
12. Mussman GM, Sahay RD, Destino L, Lossius M, Shadman KA, Walley SC. Respiratory Scores as a Tool to Reduce Bronchodilator Use in Children Hospitalized With Acute Viral Bronchiolitis. Hosp Pediatr. 2017;7:279-86.
13. Kartal O, Kartal AT. Value of neutrophil to lymphocyte and platelet to lymphocyte ratios in pneumonia. Bratisl Med J. 2017118:513–6.
14. Ergül AB, Altuner Torun Y, Uytun S, Aslaner H, Paç Kısaaslan A, Şerbetçi MC. Reduction in mean platelet volume in children with acute bronchiolitis. Turk Pediatri Ars. 2016; 51:40-5.
15. Nayak J, Hoy G, Gordon A. Influenza in Children. Cold Spring Harb Perspect Med. 2021;11:a038430.
16. Ambrożej D, Orzołek I, Makrinioti H, et al. Association of respiratory virus types with clinical features in bronchiolitis: Implications for virus testing strategies. A systematic review and meta-analysis. Paediatr Respir Rev. 202449:34-42.
17. Hemalatha R, Swetha GK, Seshacharyulu M, Radhakrishna KV. Respiratory syncitial virus in children with acute respiratory infections. Indian J Paediatr. 2010;77: 755-8.
18. Yıldırım Ş, Kaymaz N, Topaloğlu N, et al. Do we really ponder about necessity of intravenous hydration in acute bronchiolitis? Colomb Med (Cali). 2016;47:21-4.
19. Griffiths B, Riphagen S, Lillie J. Management of severe bronchiolitis: impact of NICE guidelines. Arch Dis Child. 2020;105(5):483-5.
20. Kuramochi Y, Ichinose M, Matsushita T, et al. Evaluation of the effect of inhaled β2 agonist on lung sounds of children with acute bronchiolitis. Pediatr Int. 2025;67(1):e70053.
21. Pişkin İE, Erkek Atay N, Karacan CD, İpek MŞ, Yöney A. Türkiye’deki pediatristlerin akut bronşiyolite güncel yaklaşımları. Çocuk Sağlığı ve Hastalıkları Dergisi. 2007;50:168-73.
22. Mellick L, Walsh P, Clanton C, Kalra S, McKinney S. Outpatient Medications Deimplemented by the AAP Bronchiolitis Guidelines: An Umbrella Review of Meta-Analyses. Pediatr Pulmonol. 2025;60(1):e27391.
23. Pasanen A, Karjalainen MK, Korppi M, Hallman M, Rämet M; Research Project FinnGen. Genetic Susceptibility to Acute Viral Bronchiolitis. J Infect Dis. 2025;232(2):e193-e202.
24. Castro-Rodriguez JA, Astudillo P, Puranik S, Brown MA, Custovic A, Forno E. New paradigms in acute viral bronchiolitis: Is it time to change our approach? Paediatr Respir Rev. 2025;56:29-36.
25. Republic of Turkey Ministry of Health, Board of Specialization in Medicine (TUKMOS). Pediatric Diseases Core Curriculum (v3.0). Ankara: Ministry of Health; 2023.
26. Pasanen A, Karjalainen MK, Korppi M, Hallman M, Rämet M; Research Project FinnGen. Genetic Susceptibility to Acute Viral Bronchiolitis. J Infect Dis. 2025;232(2):193-202.

**CASE REPORT FORM**

1. How old are you?
2. What is your gender?
   a. Woman
   b. Man
3. What is your medical title?
a. Pediatric resident
b. Pediatrician
c. Pediatrician, subspecialist
4. Which hospital do you work at?
a. State Hospital
b. Training and Research Hospital
c. University Hospital
d. Other
5. What is your years in the profession (year)?
6. What is the area you most frequently work in?
a. Children's outpatient clinic
b. Children's ward
c. Children's emergency room
d. Pediatric intensive care unit
e. Neonatal intensive care unit
7. How many cases of acute bronchiolitis do you see in a month?
8. After taking the history and performing the physical examination, which tests would you order to diagnose bronchiolitis? (You can select more than one option)
   a. I do not use diagnostic tests
   b. Complete blood count
   c. Acute phase reactants
   d. Blood gas
   e. Posteroanterior chest X-ray
   f. Respiratory viral pathogens
   g. Influenza rapid test
   h. Respiratory Syncytial Virus rapid test
9. Do you use the bronchiolitis score in the diagnosis and monitoring of bronchiolitis?

Yes	No

10. Do you use guidelines in the diagnosis and monitoring of bronchiolitis? Yes	No
11. Which guidelines do you use for the treatment of bronchiolitis?
12. To the emergency room What are your treatment preferences for a patient presenting with **moderate bronchiolitis ? (You can select more than one option)**
    a. Oxygen
    b. Hydration
    c. Nebulized salbutamol
    d. Nebulized epinephrine
    e. Nebulized hypertonic saline
    f. Systemic steroids
    g. HFNC
    h. nCPAP
13. To the emergency room, what are your treatment preferences for a patient presenting with **severe bronchiolitis ? (You can select more than one option)**
    a. Oxygen
    b. Hydration
    c. Nebulized salbutamol
    d. Nebulized epinephrine
    e. Nebulized hypertonic saline
    f. Systemic steroids
    g. HFNC
    h. nCPAP
14. In the children's ward, what are your treatment preferences for a patient presenting with **moderate bronchiolitis? (You can select more than one option)**
    a. Oxygen
    b. Hydration
    c. Nebulized salbutamol
    d. Nebulized epinephrine
    e. Nebulized hypertonic saline
    f. Systemic steroids
    g. HFNC
    h. nCPAP
15. In the children's ward, what are your treatment preferences for a patient presenting with **severe bronchiolitis ? (You can select more than one option)**
    a. Oxygen
    b. Hydration
    c. Nebulized salbutamol
    d. Nebulized epinephrine
    e. Nebulized hypertonic saline
    f. Systemic steroids
    g. HFNC
    h. nCPAP

**Table 1.** Demographic data of pediatricians

| Age (year±SD) (n=239) | 36.6±6.4 |
| --- | --- |
| Female (n,%) | 166 (69.5) |
| Pediatricians (n,%)  Resident physicians  General pediatrics specialists  Subspecialty specialists | 50 (20.9)  174 (72.8)  15 (6.3) |
| The type of hospital (n,%)  State Hospital  Training and Research Hospital  University Hospital  Others | 59 (24.7)  107 (44.8)  41 (17.2)  32 (13.4) |
| The years of medical practice (year) | 10.0±7.1 |
| The number of acute bronchiolitis cases/month (median, IQR) | 42 (5-40) |

**Table 2.** Comparison of Diagnostic Approaches, and Treatment Modalities Between Guideline-Adherent and Non-Adherent Pediatricians

|  | **Group 1**  **(n=143)** | **Group 2 (n=96)** | ***p*** | **Total**  **(n=239)** |
| --- | --- | --- | --- | --- |
| **Use of acute bronchiolitis scoring system (n,%)**  **Use of acute bronchiolitis guidelines (n,%)** | 66 (46.1)  143 (59.8) | 25 (25.0)  96 (40.2) | **<0.005** | 91 (38.1)  143 (59.8) |
| Use of the diagnostic tests for cases in acute bronchiolitis (n,%)  I do not use diagnostic tests  Complete blood count  Acute phase reactants  Blood gas  Posteroanterior chest X-ray  Respiratory viral pathogens  Influenza rapid test  Respiratory Syncytial Virus rapid test | 54 (37.8)  50 (34.9)  43 (30.1)  44 (30.8)  63 (44.1)  37 (25.9)  18 (12.6)  26 (18.2) | 37 (38.5)  37 (38.3)  44 (45.8)  23 (23.9)  39 (40.6)  15 (15.6)  12 (12.5)  15 (15.6) | 0.903  0.573  **0.013**  0.250  0.599  0.060  0.984  0.607 | 91 (38.1)  87 (36.4)  87 (36.4)  67 (28.0)  102 (42.7)  52 (21.8)  30 (12.6)  41 (17.2) |
| **Moderately ill in PED (n,%)**  Oxygen  Hydration  Nebulized salbutamol  Nebulized epinephrine  Nebulized hypertonic saline  Systemic steroids  HFNC  nCPAP | 113 (89.0)  111 (86.6)  79 (62.2)  11 (8.7)  17 (22.0)  27 (21.3)  18 (14.2)  15 (11.8) | 86 (76.8)  77 (68.8)  94 (83.9)  19 (16.9)  15 (43.7)  55 (49.1)  13 (11.6)  3 (2.6) | **0.012**  **<0.005**  **<0.005**  0,053  0.574  **<0.005**  0,556  **0.008** | 199 (83.3)  188 (78.7)  173 (72.4)  30 (12.6)  32 (13.4)  82 (34.3)  31 (13.0)  18 (7.5) |
| **Severely ill in PED (n,%)**  Oxygen  Hydration  Nebulized salbutamol  Nebulized epinephrine  Nebulized hypertonic saline  Systemic steroids  HFNC  nCPAP | 113 (88.9)  119 (93.7)  97 (76.4)  35 (27.6)  18 (14.2)  80 (62.9)  99 (78.0)  38 (29.9) | 102 (91.1)  100 (89.2)  99 (88.4)  33 (29.5)  22 (19.6)  90 (80.4)  48 (42.9)  15 (13.4) | 0.591  0.160  **0.016**  0.427  0.169  0.005  **<0.005**  **0.002** | 215 (90.0)  219 (91.6)  196 (82.0)  68 (28.5)  40 (16.7)  170 (71.1)  147 (61.5)  53 (22.2) |
| **Moderately ill in PW (n,%)**  Oxygen  Hydration  Nebulized salbutamol  Nebulized epinephrine  Nebulized hypertonic saline  Systemic steroids  HFNC  nCPAP | 120 (94.5)  116 (91.3)  82 (64.6)  9 (7.1)  18 (14.1)  43 (33.8)  32 (25.2)  19 (15.0) | 88 (78.6)  93 (83.0)  95 (84.9)  12 (10.7)  20 (17.9)  61 (53.5)  21 (18.8)  5 (4.5) | **<0.005**  0.053  **<0.005**  0.323  0.437  **<0.005**  0.231  **0.007** | 208 (87.0)  209 (87.4)  177 (74.1)  21 (8.8)  38 (15.9)  104 (43.5)  53 (22.2)  24 (10.0) |
| **Severely ill in PW (n,%)**  Oxygen  Hydration  Nebulized salbutamol  Nebulized epinephrine  Nebulized hypertonic saline  Systemic steroids  HFNC  nCPAP | 127 (100)  119 (93.7)  98 (77.2)  34 (26.8)  99 (78.0)  74 (58.3)  99 (77.9)  51 (40.1) | 112 (100)  106 (94.6)  101 (90.2)  29 (25.8)  62 (55.4)  93 (83.0)  62 (55.4)  22 (19.6) | **-**  0.757  **0.007**  0.878  **<0.005**  **<0.005**  **<0.005**  **0.001** | 239 (100)  225 (94.1)  199 (83.3)  63 (26.4)  46 (19.2)  167 (69.9)  161 (67.4)  73 (30.5) |

HFNC: High Flow Nasal Oxygenation, nCPAP: Nasally applied continuous positive airway pressure, PED: Pediatric emergency department, PW: Pediatric ward

**Table 3.** Comparison of Diagnostic Approaches, Laboratory Tests, and Treatment Modalities Between Resident Pediatrics and Pediatric Specialists

|  | **Resident pediatrics (n=50)** | **Pediatric specialists (n=189)** | ***p*** |
| --- | --- | --- | --- |
| **Use of acute bronchiolitis scoring system (n,%)**  **Use of acute bronchiolitis guidelines (n,%)** | 20 (40.0)  40 (80.0) | 71 (37.5)  103 (54.5) | 0.753  0.001 |
| Use of the diagnostic tests for cases in acute bronchiolitis (n,%)  I do not use diagnostic tests  Complete blood count  Acute phase reactants  Blood gas  Posteroanterior chest X-ray  Respiratory viral pathogens  Influenza rapid test  Respiratory Syncytial Virus rapid test | 18 (36.0)  18 (36.0)  17 (34.0)  16 (32.0)  27 (54.0)  18 (36.0)  6 (12.0)  9 (18.0) | 73 (38.6)  68 (35.9)  70 (37.0)  51 (26.9)  75 (39.7)  34 (17.9)  24 (12.7)  32 (16.9) | 0.704  0.792  0.691  0.483  0.069  **0.006**  0.894  0.859 |
| **Moderately ill in PED (n,%)**  Oxygen  Hydration  Nebulized salbutamol  Nebulized epinephrine  Nebulized hypertonic saline  Systemic steroids  HFNC  nCPAP | 43 (86.0)  41 (82.0)  43 (86.0)  2 (4.0)  5 (10.0)  15 (30.0)  4 (8.0)  7 (14.0) | 156 (84.2)  147 (77.8)  130 (68.8)  28 (14.8)  27 (14.3)  67 (35.4)  27 (14.3)  11 (5.8) | 0.560  0.517  **0.015**  **0.040**  0.429  0.470  0.239  0.051 |
| **Severely ill in PED (n,%)**  Oxygen  Hydration  Nebulized salbutamol  Nebulized epinephrine  Nebulized hypertonic saline  Systemic steroids  HFNC  nCPAP | 44 (88.0)  46 (92.0)  34 (68.0)  8 (16.0)  9 (18.0)  33 (66.0)  39 (78.0)  9 (18.0) | 171 (90.5)  173 (91.5)  162 (85.7)  60 (31.7)  31 (16.4)  137 (72.5)  108 (57.1)  44 (23.3) | 0.604  0.916  **0.004**  **0.028**  0.788  0.554  **0.007**  **0.424** |
| **Moderately ill in PW (n,%)**  Oxygen  Hydration  Nebulized salbutamol  Nebulized epinephrine  Nebulized hypertonic saline  Systemic steroids  HFNC  nCPAP | 41 (82.0)  45 (90.0)  34 (68.0)  1 (2.0)  11 (22.0)  15 (30.0)  14 (28.0)  10 (20.0) | 167 (88.4)  164 (86.7)  143 (75.7)  20 (10.6)  27 (14.3)  89 (47.1)  39 (20.6)  14 (7.4) | 0.234  0.540  0.272  0.057  0.185  **0.030**  0.265  **0.008** |
| **Severely ill in PW (n,%)**  Oxygen  Hydration  Nebulized salbutamol  Nebulized epinephrine  Nebulized hypertonic saline  Systemic steroids  HFNC  nCPAP | 50 (100)  46 (92.0)  38 (76.0)  6 (12.0)  13 (26.0)  30 (60.0)  40 (80.0)  17 (34.0) | 189 (100)  179 (94.7)  161 (85.1)  57 (30.2)  33 (17.5)  137 (72.3)  121 (64.0)  56 (29.6) | -  0.468  0.122  **0.010**  0.173  0.087  **0.032**  0.551 |

HFNC: High Flow Nasal Oxygenation, nCPAP: Nasally applied continuous positive airway pressure, PED: Pediatric emergency department, PW: Pediatric ward

**Table 4.** Multivariable Logistic Regression Analysis of Factors Associated with Guideline Non-Adherence among Pediatricians

| **Variables in the Equation** | **B** | **S.E.** | **Wald** | **df** | **p-value (Sig.)** | **Odds Ratio [Exp (B)]** | **95% Confidence Interval for Exp(B) (Lower – Upper)** |
| --- | --- | --- | --- | --- | --- | --- | --- |
| **Hospital Type** |  |  | 8.154 | 3 | **0.043** |  |  |
| State Hospital | 1.795 | 0.878 | 4.185 | 1 | **0.041** | 6.021 | 1.078 – 33.627 |
| University Hospital | 2.814 | 1.026 | 7.529 | 1 | **0.006** | 16.677 | 2.234 – 124.471 |
| **Acute phase reactants** | -1.498 | 0.643 | 5.432 | 1 | **0.020** | 0.224 | 0.063 – 0.788 |
| **PED-Moderate: Nebulized Salbutamol** | -1.940 | 0.698 | 7.725 | 1 | **0.005** | 0.144 | 0.037 – 0.564 |
| **PED-Moderate: HFNC** | -2.210 | 1.073 | 4.239 | 1 | **0.040** | 0.110 | 0.013 – 0.899 |
| **PED-Moderate: nCPAP** | 4.588 | 2.057 | 4.973 | 1 | **0.026** | 98.264 | 1.743 – 5539.146 |
| **PW-Moderate: Hydration Support** | 5.917 | 1.708 | 12.008 | 1 | **0.001** | 371.291 | 13.069 – 10548.319 |
| **PW-Moderate: Nebulized Epinephrine** | -3.420 | 1.487 | 5.291 | 1 | **0.021** | 0.033 | 0.002 – 0.603 |
| **PW-Severe: Hydration Support** | -6.237 | 1.934 | 10.404 | 1 | **0.001** | 0.002 | 0.000 – 0.087 |
| **PW-Severe: Nebulized Salbutamol** | -2.396 | 0.947 | 6.396 | 1 | **0.011** | 0.091 | 0.014 – 0.583 |
| **PW-Severe: Nebulized Hypertonic Saline** | 3.286 | 1.431 | 5.272 | 1 | **0.022** | 26.732 | 1.618 – 441.756 |
| **PW-Severe: HFNC** | 2.036 | 0.771 | 6.967 | 1 | **0.008** | 7.661 | 1.689 – 34.747 |
| **PW-Severe: nCPAP** | 1.816 | 0.807 | 5.070 | 1 | **0.024** | 6.148 | 1.265 – 29.868 |

HFNC: High Flow Nasal Oxygenation, nCPAP: Nasally applied continuous positive airway pressure, PED: Pediatric emergency department, PW: Pediatric ward
